# Dysregulated expression of antioxidant enzymes in polyethylene particle-induced periprosthetic inflammation and osteolysis

**DOI:** 10.1371/journal.pone.0202501

**Published:** 2018-08-20

**Authors:** Kuo-Ti Peng, Meng-Hsueh Tsai, Chiang-Wen Lee, Yao-Chang Chiang, Pei-Chun Chen, Chun-Chieh Chen, Chih-Hsiang Chang, Hsin-Nung Shih, Pey-Jium Chang

**Affiliations:** 1 Department of Orthopedic Surgery, Chang Gung Memorial Hospital, Chiayi, Taiwan; 2 College of Medicine, Chang-Gung University, Taoyuan, Taiwan; 3 Division of Basic Medical Sciences, Department of Nursing, and Chronic Diseases and Health Promotion Research Center, Chang Gung Institute of Technology, Chiayi, Taiwan; 4 Center for Drug Abuse and Addiction, China Medical University Hospital, Taichung, Taiwan; 5 Department of Orthopedic Surgery, Chang Gung Memorial Hospital, Linkou, Taiwan; 6 Graduate Institute of Clinical Medical Sciences, College of Medicine, Chang-Gung University, Taoyuan, Taiwan; 7 Department of Nephrology, Chang-Gung Memorial Hospital, Chiayi, Taiwan; Alexandria University, EGYPT

## Abstract

Small wear particles (0.1–10 μm) in total joint replacement are generally considered as the major causative agent leading to periprosthetic inflammation and osteolysis. However, little is known about the roles of larger wear particles (10–100 μm) in periprosthetic inflammation and osteolysis. Additionally, although ample studies demonstrated that increased oxidative stress is critically involved in particle-induced inflammation and osteolysis, detailed changes in antioxidant enzymes expression in the disease development remain largely unclear. Herein, we used a rat knee prosthesis model to assess effects of polyethylene (PE) particles (20–60 μm) on the levels of oxidative stress markers such as malondialdehyde (MDA) and total antioxidant capacity (TAC) in blood plasma, and on the expression profiles of antioxidant enzymes in knee joint tissues. In combination with a forced-exercise intervention for all surgical rats, we found that the rat groups treated with both artificial joint and PE particles exhibited higher MDA levels and lower TAC levels, together with lower levels of physical activity and higher levels of inflammatory markers, than the sham group and the groups receiving artificial joint or PE particles alone at weeks 20–24 post-operatively. Dose-response relationships between the exposure to PE particles and the induction of oxidative stress and inflammation were also observed in the artificial joint/PE groups. Under such conditions, we unexpectedly found that most of antioxidant enzymes displayed pronounced up-regulation, with concomitant induction of inflammatory and osteoclast-inducing factors (including IL-1β, NF-κB and RANKL), in the artificial joint/PE groups as compared to the sham, artificial joint only, or PE only group. Only a few antioxidant enzymes including SOD2 and GPx2 showed down-regulation. Collectively, our findings demonstrate that implantation of artificial joint along with large PE particles synergistically trigger the induction of oxidative stress; however, down-regulation of many antioxidant enzymes may not necessarily occur during the disease development.

## Introduction

Total hip replacement and total knee replacement are commonly used to treat a variety of joint disease patients. Aseptic loosening is the major cause of complications after total joint replacement and the revision rate is around 8 to 15% in hip and knee arthroplasties [1–3]. There are several theories about the causes of aseptic loosening [4]. One of the main theories is linked to wear-generated debris [5, 6]. It is well accepted that phagocytosis of wear particles by macrophages triggers the release of various inflammatory mediators that lead to the recruitment and differentiation of bone-resorbing osteoclasts during the aseptic inflammatory process [5, 7]. Due to the prevalent use of metal-on-polyethylene prosthesis, wear-generated polyethylene (PE) particles are considered as the major player in eliciting inflammatory response and osteoclastogenesis [5].

Reactive oxygen species (ROS) are known as important stimulators of inflammation. Numerous studies have revealed that the ROS-mediated oxidative stress is critically involved in wear particle-induced osteolysis [8–10]. High levels of oxidative stress biomarkers have been observed in periprosthetic tissues of patients with aseptic implant loosening [11], and in a variety of cultured cell types after exposure to wear particles [10, 12–14]. In addition to the sustained stimulation of chronic inflammation, increased ROS production is also required for RANKL-mediated osteoclast activation and differentiation [15–17]. In the cellular system, dynamic changes in ROS levels mainly depend on actions of ROS-producing enzymes (e.g. NADPH oxidase) and various antioxidant proteins such as superoxide dismutases (SODs), catalase, glutathione peroxidases (GPxs), transferrin and transthyretin (TTR) [18, 19]. Therefore, the use of specific antioxidants or inhibitors of NADPH oxidase has been reported to reduce cytokine- or particle-mediated ROS production and osteoclastic bone resorption *in vitro* and *in vivo* [9, 20]. In our previous study, we also found that several antioxidant enzymes were aberrantly expressed in synovial fluid of patients with aseptic loosening, suggesting that dysregulation of ROS-related proteins may be critically associated with the development of aseptic loosening [21].

Although multiple lines of evidence support the involvement of oxidative stress in particle-induced periprosthetic inflammation and osteolysis, several questions have left unanswered. These unanswered questions include (i) whether wear particles alone, especially with particle sizes that are larger than the optimum size (0.1–10 μm) for phagocytosis by macrophages, are sufficient to promote ROS accumulation and the subsequent inflammation or osteoclastogenesis *in vivo*, (ii) whether oxidative stress markers measured in blood samples could reflect disease progression of particle-induced periprosthetic osteolysis, and (iii) whether down-regulation of antioxidant enzymes is a common event in the progression of wear particle-induced inflammation and osteolysis.

In this study, we attempted to use a rat knee prosthesis model to address the above questions. Since wear-generated PE particles in the size range of 0.1–10 μm have long been considered the major contributor to inflammation and osteoclast differentiation [22–25], the present study aimed to investigate the potential association between large PE particles (20–60 μm) and oxidative stress, inflammation or osteolysis in our rat knee model.

## Materials and methods

### Animal model and experimental grouping

**Fig 1:**
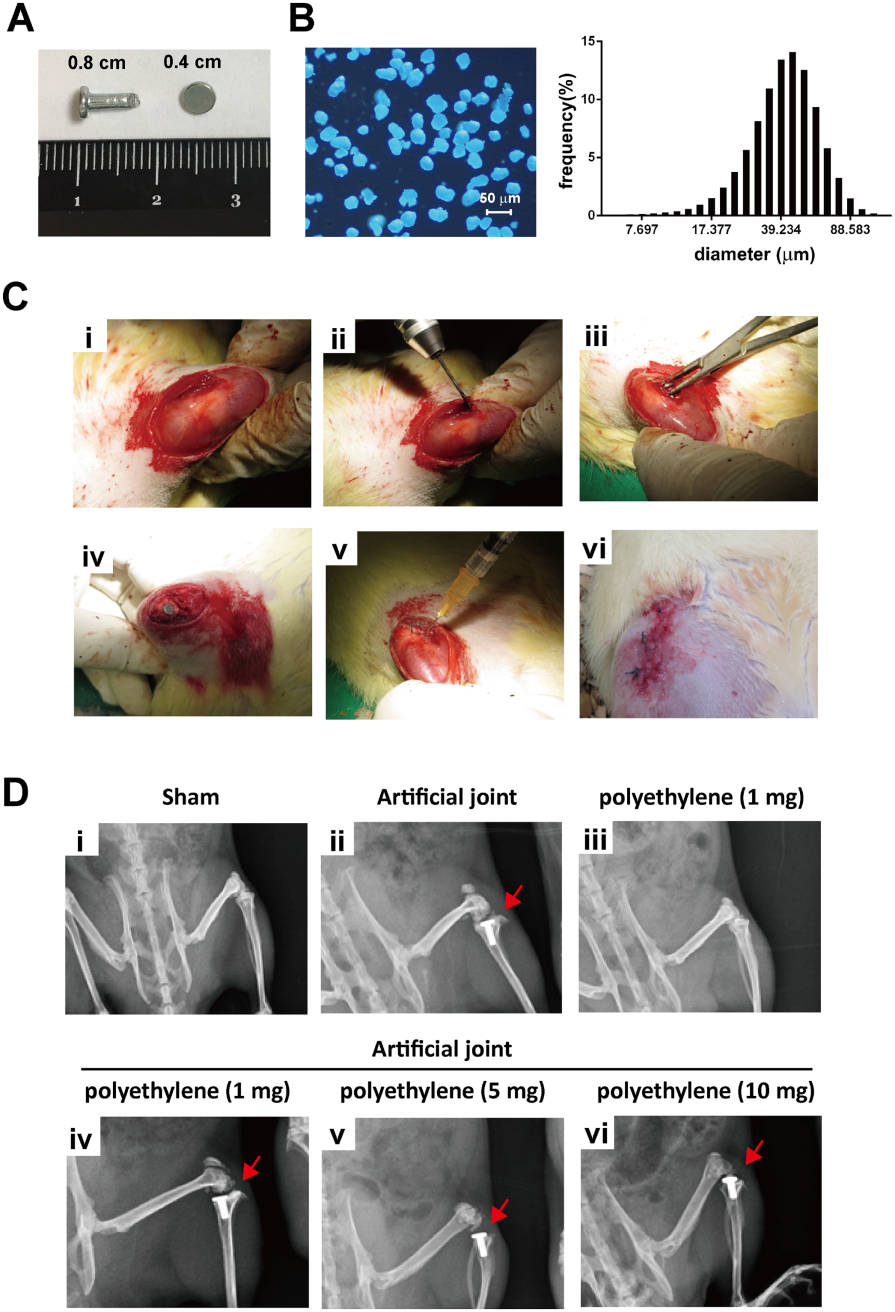
Implantation of an artificial knee joint and PE particles in rats. (A) An artificial knee joint (length 0.8 cm, diameter 0.4 cm for the tibial platform) with smooth surface was used in the study. (B) The size distributions of PE particles were examined by optical microscopy (left panel) and by Horiba Laser Diffraction Particle Size Analyser LA-950 (right panel). (C) Surgical procedures for implantation of an artificial prosthesis or/and PE particles in rat knees. These procedures include (i) skin and soft tissue incision, (ii) creation of an intramedullary canal in the right tibia, (iii) implantation of an artificial joint, (iv) fixation of the artificial joint using a cementing method, (v) injection of PE particle-containing hyaluronate solution, and (vi) wound closure. (D) Representative radiograph images of the proximal femur in all surgical groups.

Wistar male rats of 12 week-old (weighting 350–400 gram) obtained from Bio-LASCO Co. Ltd (Taipei, Taiwan) were used in the study. The rats were divided into six groups, including the sham surgery group and five different treated groups (n = 5 for each group). The five experimental groups were the following: (i) the group that received a titanium artificial joint only; (ii) the group that received polyethylene (PE) particles (1 mg) only; (iii to v) the groups that included implantation of an artificial joint along with various amounts (1, 5, and 10 mg) of PE particles. All rats were anesthetized with ketamine (2 mg/kg) and an anterior knee incision was made by scalpel. For all five experimental groups, the anterior and posterior cruciate ligaments were first removed by knife, and then a tibial intramedullary canal with a depth of approximately 1.0 cm was created using a 2-mm twist drill. After resection of the tibial plateau using a 1.27 mm saw blade, a custom-made titanium artificial tibial prosthesis (Fig 1A) was implanted and fixed with bone cement (Stryker^®^ Howmedica OSTEONICS, USA) in the assigned groups (artificial joint replacement groups). The artificial tibial prosthesis (0.2 cm in diameter and 0.8 cm in length) contains a tibial platform of 0.4 cm in diameter (Fig 1A). Subsequently, the medial parapatellar arthrotomy wound was repaired with sutures. Ultra-high-molecular-weight PE particles with the size range from 20 to 60 μm (Polyethylene, #434272; 40–48 μm particle size; SIGMA–ALDRICH, USA) were prepared in different concentrations (1, 5 and 10 mg/ml) in Sterile Sodium Hyaluronate Solution (10 mg/ml) (HYAJOINT ^®^, Taiwan). The size and distribution of ultra-high-molecular-weight PE particles used in the study were re-evaluated by optical microscopy and by Horiba Laser Diffraction Particle Size Analyser LA-950 (Fig 1B). One milliliter of hyaluronate solutions with or without PE particles were then injected near the implanted site of prostheses using a sterile syringe. After surgical intervention, the wound was closed and the wound area was coated with antibiotics ointment. All experimental protocols for animals were approved by the Institutional Animal Care and Use Committee of the Chang Gung Memorial Hospital (No. 2015030602), and were performed in accordance with the Animal Protection Law by the Council of Agriculture, Executive Yuan (R.O.C.) and the guideline of National Research Council (U.S.A.) for the care and use of laboratory animals.

### Wheel-running exercise and assessment of the physical activity level

After a 4-week post-surgical recovery, all surgical rats in each group were subjected to daily forced wheel-running exercises using RAT 4CH FORCED EXERCISE system (Diagnostic & Research Instruments Co, Taiwan) (S1 Fig). The wheel-running system is constructed with 32 cm in diameter, 8.5 cm in width, and 0.7 cm in the roller space. The distance of the forced wheel-running exercise was set as 500 meters per day (usually done within 90–120 minutes), five days per week for 20 weeks. The time duration required to finish the wheel-running course was routinely recorded to assess levels of the physical activity.

### Collection and preparation of blood samples and knee joint tissues

Blood samples (1 ml) were collected from rat tail veins before operation and at different postoperative time points (4, 8, 12, 16, 20 and 24 weeks). After centrifugation at 5000 xg for five minutes at 4°C, the top plasma layer was transferred to a new tube and stored at –80°C. Rats were sacrificed 24 weeks after operation, and knee joint tissues were taken and stored at –80°C.

### Analysis of malondialdehyde (MDA) in blood plasma

Levels of plasma MDA in the collected blood samples were measured by Lipid Peroxidation Assay kit (K739-100; MDA; Bio Vision, USA) according to the manufacturer’s instructions. Briefly, 20 μl of blood plasma samples were prepared, and mixed with thiobarbituric acid (TBA) in the glacial acetic acid medium. The MDS-TBA adduct in the reaction was quantified using a plate reader at a wavelength of 532 nm.

### Measurement of total antioxidant capacity (TAC) of blood plasma

Antioxidant assay kit (709001; Cayman Chemical, USA) was used to assess total antioxidant capacity (TAC) in blood samples. In the assay, a standard curve that included different concentrations of Trolox, a water-soluble analog of vitamin E, was prepared. Concentrations of TAC of plasma samples were measured using a plate reader at a wavelength of 405 nm according to the manufacturer’s instructions.

### Hematoxylin and eosin (H&E) staining and immunohistochemistry

Serial paraffin sections of knee joint tissues were cut at 5-μm thickness and were used for H&E staining and for immunohistochemical staining. For immunohistochemical staining of IL-1β, tissue sections (5-μm) were incubated with anti-IL-1β antibody (200 dilution; P420B; Thermo Fisher Scientific, USA) overnight at 4 °C, followed by using the Mouse/Rabbit Probe HRP Labeling kit (BioTnA, Kaohsiung, Taiwan). All stained slides were digitized using an Motic Easyscan Digital Slide Scanner (Motic Hong Kong Limited, Hong Kong, China). Motic Easyscan whole-slide images were viewed with DSAssistant and EasyScanner software at Litzung Biotechnology INC (Kaohsiung, Taiwan). Inflammatory index scores were graded according to inflammatory cell infiltration assessed on H&E staining (score 0, no evidence of inflammation; score 1, low; score 2, moderate; score 3, high; score 4, severe) [26]. Staining scores of IL-1β in tissue sections were evaluated by multiplying the percentage of positive cells (P) by the staining intensity score (I), as proposed by Krajewska et al. and Shen et al. [27, 28].

### Western blot analysis

Rat knee joint tissues were homogenized and lysed in a protein extraction reagent (Tissue protein extraction reagent, Pierce, Rockford, US). Western blot analysis was performed as described previously [29]. The primary antibodies to transferrin (17435-1-AP; Proteintech), catalase (ab209211; Abcam), SOD1 (ab16831; Abcam), SOD2 (ab68155; Abcam), SOD3 (ab21974; Abcam), glutathione peroxidase 1 (ab59546; Abcam), glutathione peroxidase 2 (MAB5470; R&D Systems), transthyretin (ab9015; Abcam), NF-κB (8242S; Cell Signaling), IL-1β (ab9722; Abcam), RANKL (sc-9073; Santa Cruz), and GAPDH (sc-20357; Santa Cruz) were obtained commercially. After extensive washing, appropriate secondary antibodies were used in the experiments, including anti-rabbit IgG antibody conjugated with horseradish peroxidase (7074S; Cell Signaling), anti-mouse IgG antibody conjugated with horseradish peroxidase (7076P2; Cell Signaling), or anti-sheep IgG antibody conjugated with horseradish peroxidase (313-035-003; Jackson ImmunoResearch).

### Statistics

All results were expressed as mean ± standard deviation (SD). Statistical significance was determined by one-way analysis of variance (ANOVA) coupled with a Tukey’s post hoc test. A *P* value < 0.05 was considered as statistically significant. Statistical analyses were performed using GraphPad Prism 5 (Graph-Pad, La Jolla, CA, USA) software.

## Results

### Animal model and study design

The study contains six groups, including the sham group and five experimental groups that received an artificial knee joint (Fig 1A), PE particles (1 mg), or the combination of an artificial knee joint and different amounts of PE particles (1, 5 and 10 mg). It is worth noting that PE particles used in the study are 20–60 μm in size (Fig 1B). The surgical procedures are outlined in Fig 1C, and representative radiograph images of the implanted joint are shown in Fig 1D. To mimic chronic medical conditions, all surgical rats in different groups were additionally subjected to daily forced wheel-running exercises, which started at 4 weeks after surgery, for up to 20 weeks (a total duration of 24 weeks).

### Evaluation of oxidative stress markers in blood plasma of all treatment groups

**Fig 2:**
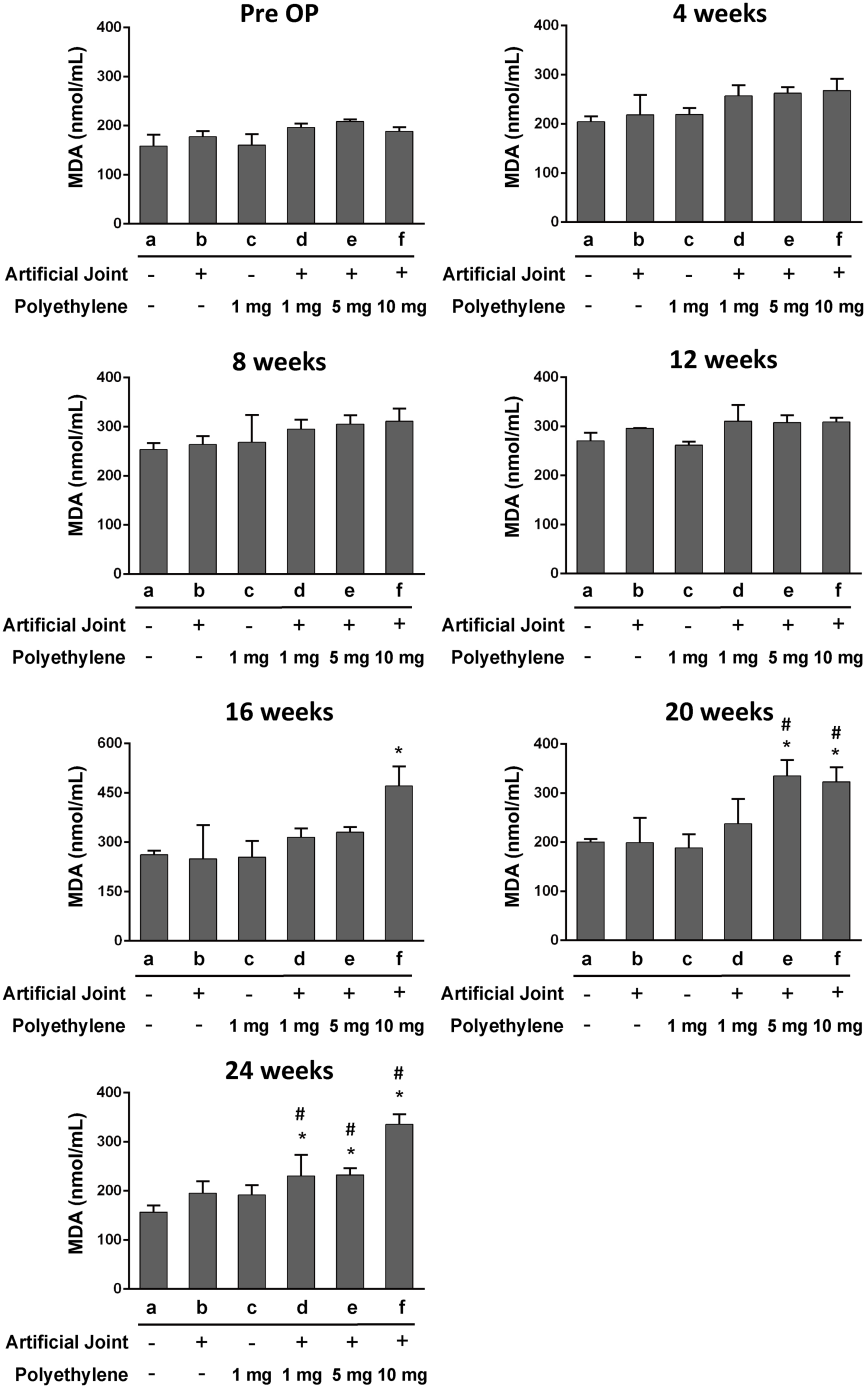
Levels of malondiadehyde (MDA) in blood plasma of all six groups before and after surgery. The concentrations of plasma MDA in different treated groups were measured at pre-operation (Pre-OP), and at 4, 8, 12, 16, 20, and 24 weeks after operation. All data are expressed as mean ± SD (n = 5 for each group). *, *P* < 0.05, for results compared to those of the sham group; #, *P* < 0.05, for results compared to those of the group receiving an artificial knee joint only.

**Fig 3:**
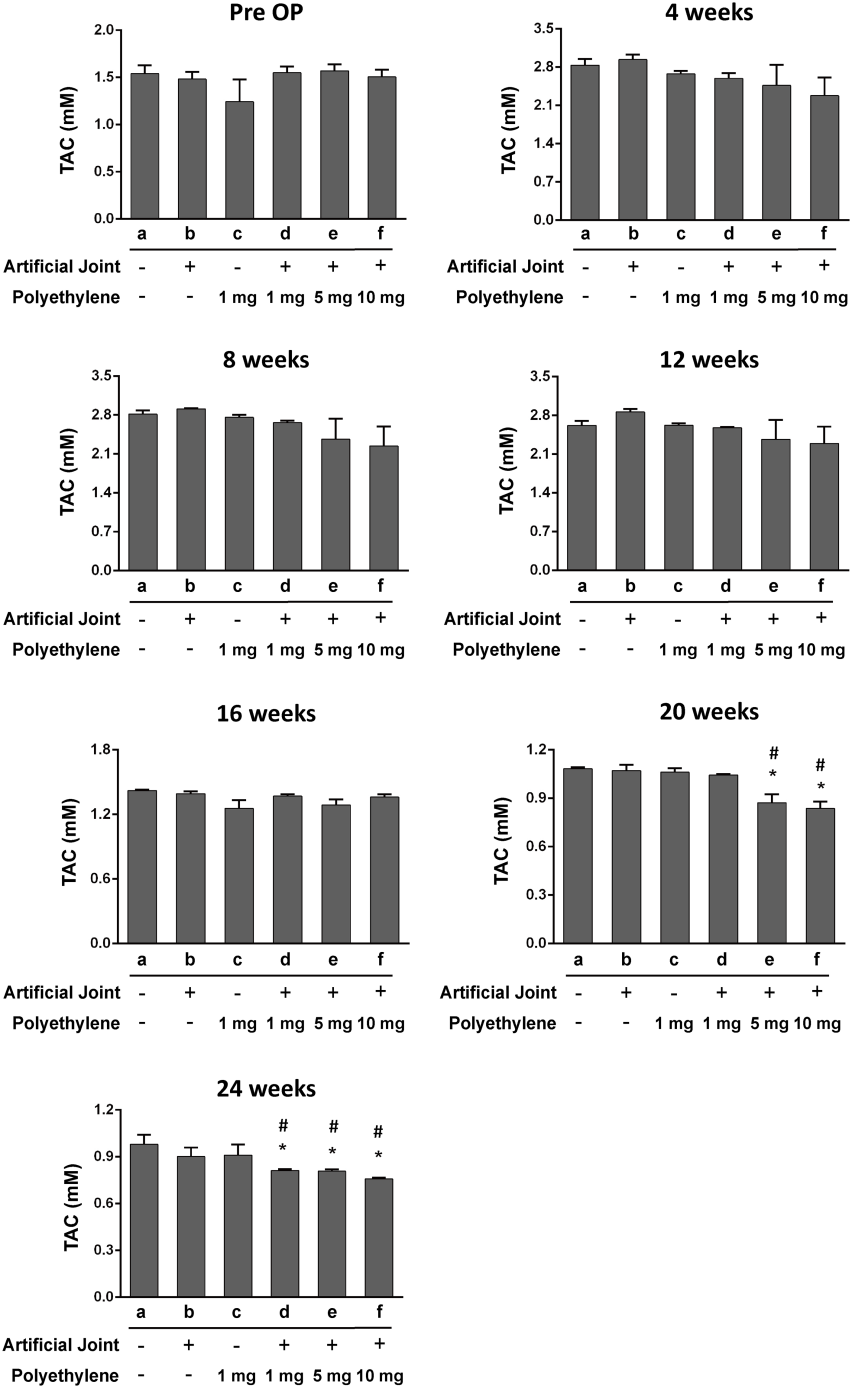
Levels of total antioxidant capacity (TAC) in blood plasma of all six groups before and after surgery. The concentrations of plasma TAC in different treated groups were evaluated at pre-operation (Pre-OP), and at 4, 8, 12, 16, 20, and 24 weeks after operation. All data are expressed as mean ± SD (n = 5 for each group). *, *P* < 0.05, for results compared to those of the sham group; #, *P* < 0.05, for results compared to those of the group receiving an artificial knee joint only.

To determine whether excessive ROS production (oxidative stress) could be detected in these treated groups, we assessed levels of MDA and TAC, two oxidative stress markers, in blood plasma. During the time period of the study, we did not find significant changes in levels of MDA and TAC between the sham group and the groups that received either artificial joint or PE particles alone (Figs 2 and 3). However, during 20–24 weeks after operation, increased MDA levels in parallel with decreased TAC levels were significantly detected in the groups receiving the combination of artificial joint and PE particles compared to the sham group (Figs 2 and 3). Notably, increasing amounts of PE particles in the artificial joint implantation groups appeared to show higher MDA levels and lower TAC levels (Figs 2 and 3), suggesting a dose-response relationship exists between the exposure to PE particles in local joint tissues and the changes of oxidative stress markers in blood.

### Assessment of physical activity levels in all treatment groups

**Fig 4:**
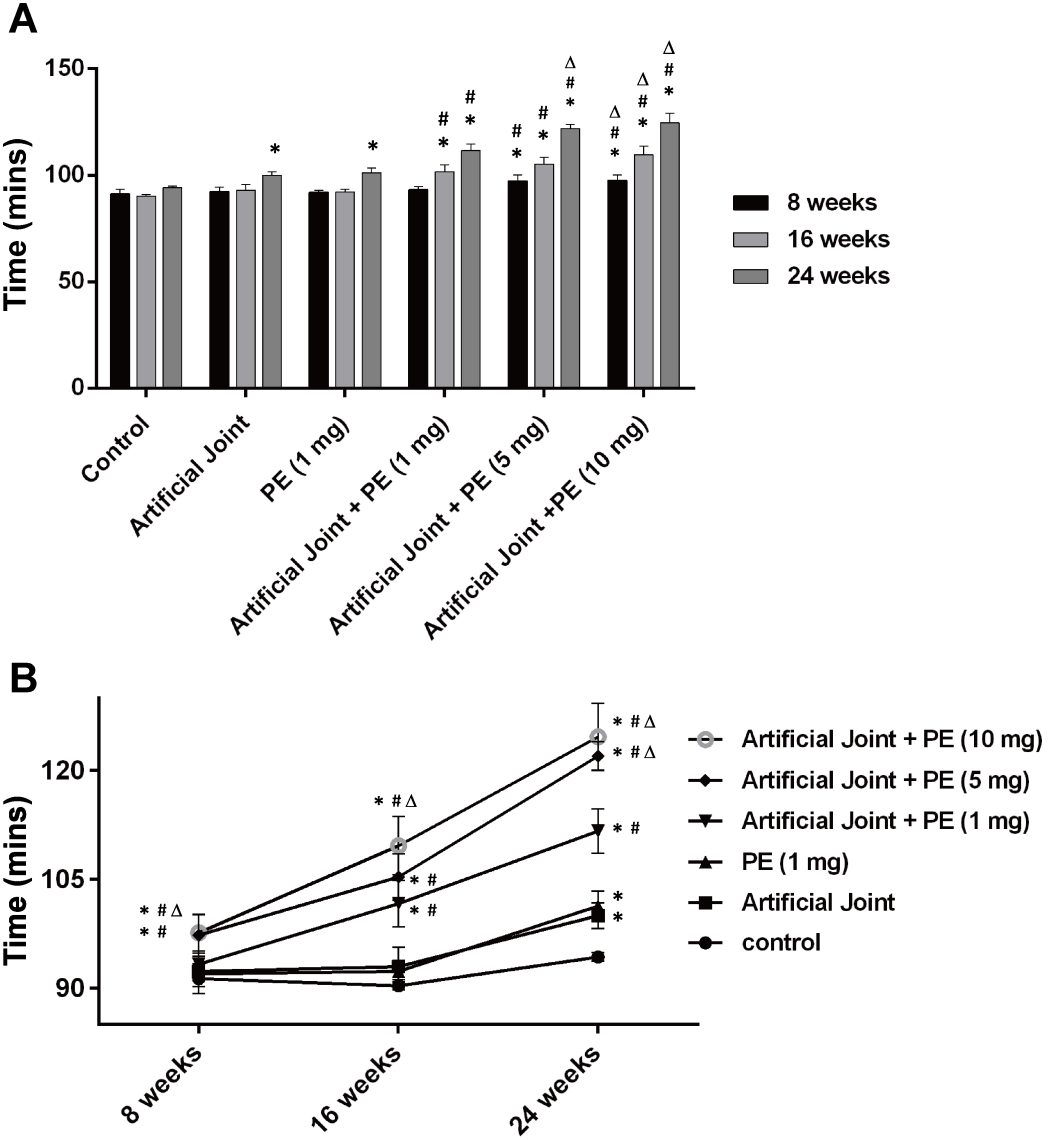
Assessment of the wheel-running performance in all treated groups after surgery. The average time duration to complete the assigned exercise (500 meters) was measured in all six groups at 8, 16, 24 weeks after operation (n = 5 for each group). All data in bar graphs (A) and line graphs (B) are expressed as mean ± SD. *, *P* < 0.05, for results compared to those of the sham group; #, *P* < 0.05, for results compared to those of the group receiving an artificial knee joint only; Δ, *P* < 0.05, for results compared to those of the group receiving the combination of an artificial knee joint and 1mg of PE particles.

As mentioned above, implantation of both artificial joint and PE particles substantially elicited systemic oxidative stress 20–24 weeks after surgery. To study the potential association between oxidative stress and disease severity, the wheel running was used to assess physical activity levels in all groups. In the sham group, the average time duration required to run 500 meters in wheels was 91, 90 and 94 min at 8, 16 and 24 weeks after surgery, respectively (Fig 4A and 4B). As compared to the sham group, longer time durations were required to complete the running course for all five experimental groups at 24 weeks after surgery (Fig 4A and 4B). Among these different experimental groups, we further found that the three artificial joint/PE groups spent more time finishing the wheel-running course than the groups receiving artificial joint or PE particles only (Fig 4A and 4B). Furthermore, PE particles appeared to act in a dose-dependent manner to increase the time duration of the running course in these artificial joint/PE groups (Fig 4A and 4B). Notably, no significant differences in body weight among these groups were observed during the experimental period (S2 Fig). These results suggested that levels of physical activity assessed by wheel running in all treatment groups were closely correlated with levels of oxidative stress markers detected in the blood.

### Histological analysis of chronic inflammation in knee joint tissues of all treatment groups

**Fig 5:**
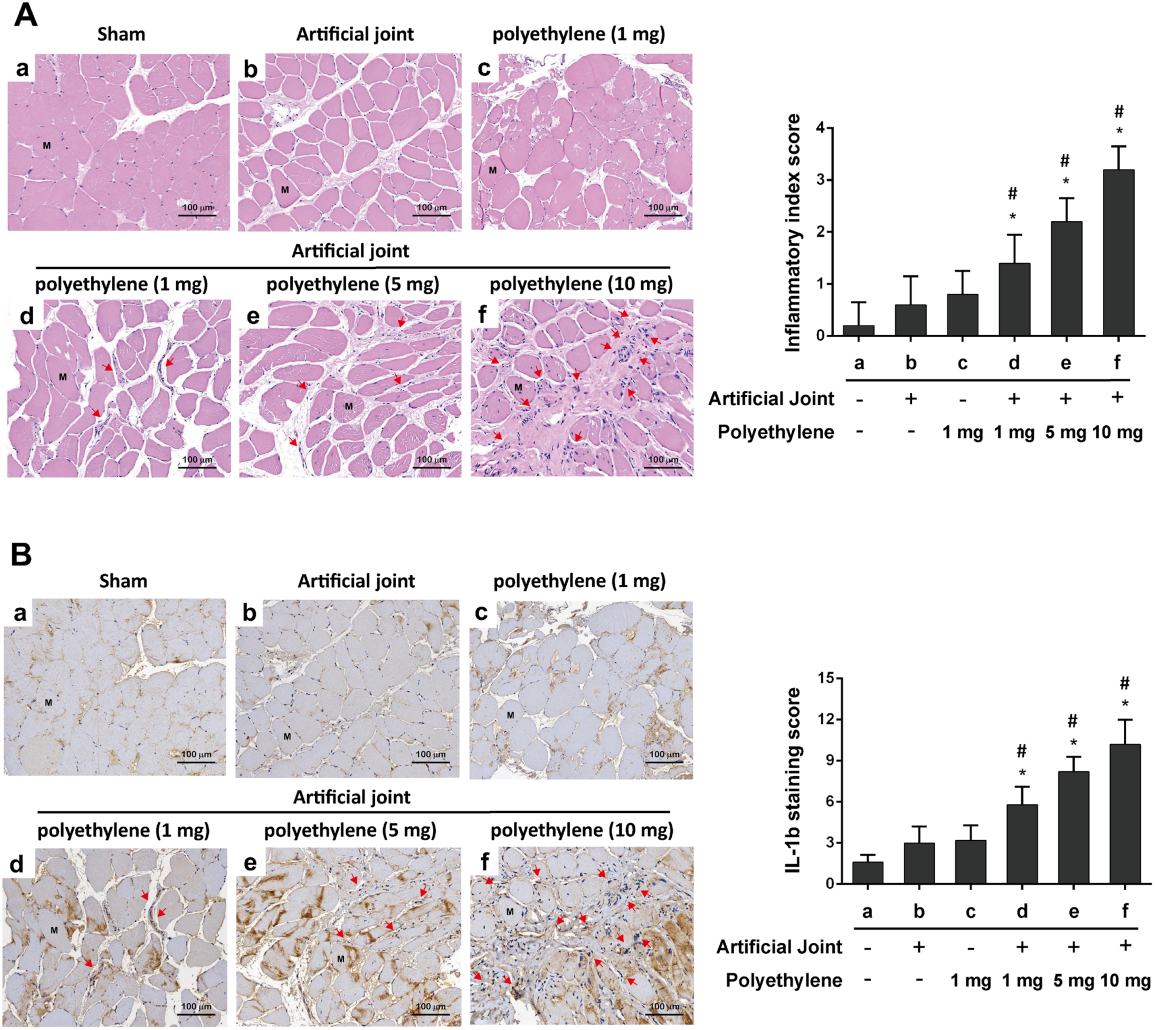
Evaluation of chronic inflammation in knee joint tissues of all treated groups at 24-week post-operation. (A) H&E evaluation of knee joint tissue samples. Tissues sections of knee joints from the indicated treated groups were stained with H&E. The inflammatory index was evaluated based on levels of inflammatory cell infiltration in knee joint tissues (n = 3 for each group). Arrows indicate infiltrating mononuclear immune cells. M, muscle cells (B) Histological evaluation of IL-1β staining in knee joint tissues. Tissues sections were stained with IL-1β (n = 3 for each group) and IL-1β-specific staining was scored as described in Materials and Methods. Arrows indicate IL-1β-positive immune cells. M, muscle cells; *, *P* < 0.05, compared to the sham group; #, *P* < 0.05, compared to the group receiving an artificial knee joint only. Bar, 100 μm.

To further evaluate and compare chronic inflammatory patterns among different treated groups, histological staining was carried out using knee joint tissues obtained from 24-week post-operative animals. Sample sections were subjected to H&E staining as well as staining with anti-IL-1β. When compared to the sham group, we did not detect a significant increase of inflammatory cell infiltration (Fig 5A) or IL-1β staining (Fig 5B) in knee joint tissues of the rat groups that received an artificial joint or PE particle alone. However, the artificial joint/PE groups significantly displayed increased numbers of infiltrating mononuclear immune cells and elevated levels of IL-1β in knee joint tissues (Fig 5A and 5B) as compare to the sham group or the groups receiving artificial joint or PE particles only. These results indicated that inflammatory responses were induced mainly in the artificial joint/PE groups.

### Expression of antioxidant proteins and osteoclast-inducing factors in knee joint tissues of all treatment groups

**Fig 6:**
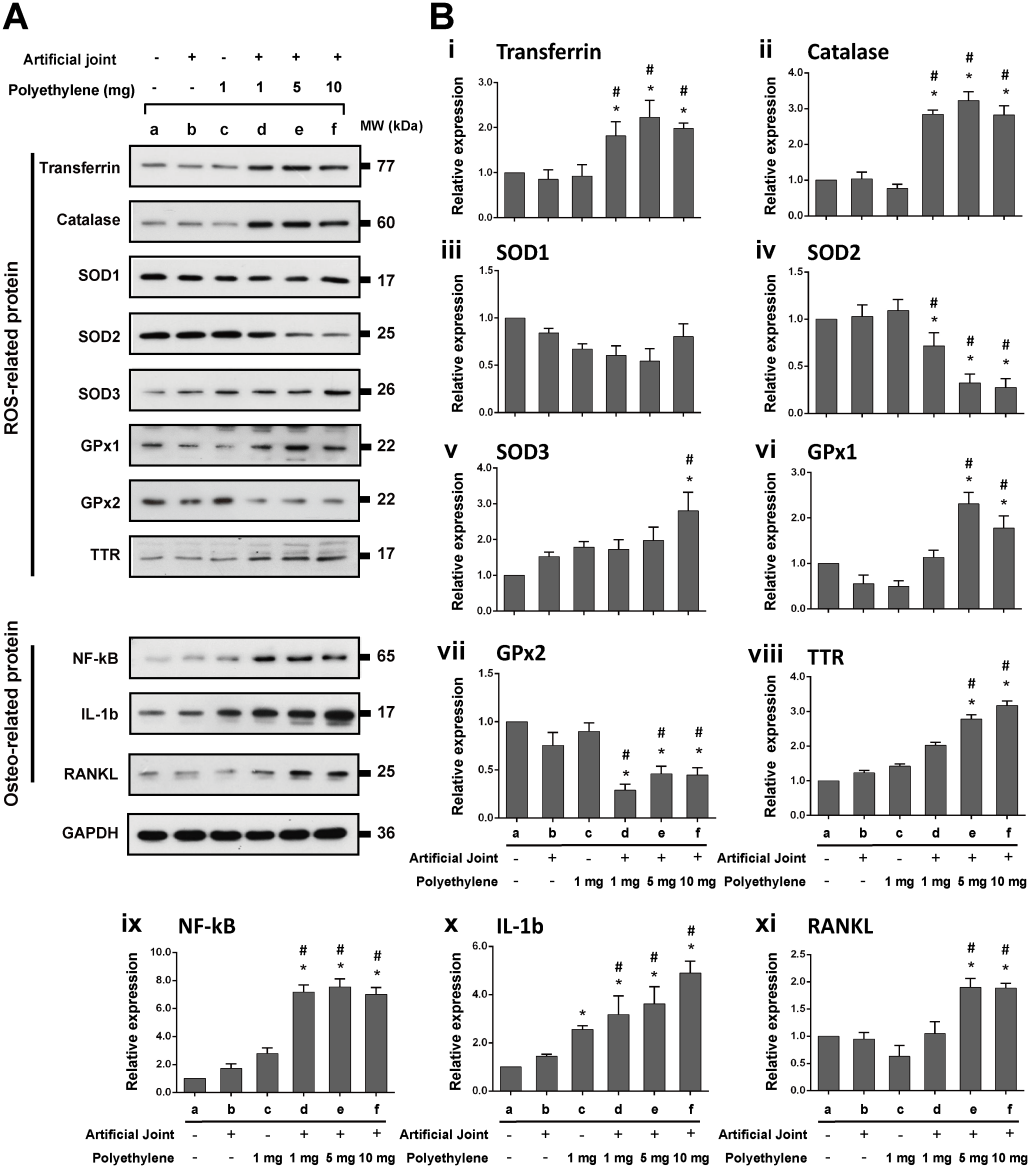
Expression of various ROS-related proteins, pro-inflammatory factors, and an osteoclast differentiation factor RANKL in tissue samples of all treated groups at 24 weeks after surgery. (A) Representative Western blot analysis of transferrin, catalase, SOD1, SOD2, SOD3, GPx1, GPx2, TTR, NF-κB, IL-1β and RANKL in different groups. GAPDH was used as internal control. (B) Densitometric quantification of Western blot analysis for all tested proteins normalized to GAPDH (n = 3 for each group). *, *P* < 0.05, for results compared to those of the sham group; #, *P* < 0.05, for results compared to those of the group receiving an artificial knee joint only.

We next examined the protein expression of different antioxidant proteins including transferrin, catalase, SOD1, SOD2, SOD3, GPx1, GPx2 and transthyretin (TTR), pro-inflammatory factors including NF-κB and IL-1β, as well as the osteoclast-inducing factor RANKL in knee joint tissue samples collected at 24-week post-operation (Fig 6). When compared to the sham group, we did not detect significant differences in the expression profiles of all tested antioxidant proteins in the groups receiving an artificial joint or PE particles alone, which was consistent with the unchanged MDA and TCA levels detected in these groups. However, differential up- or down-regulation of various antioxidant proteins was markedly detected in the artificial joint/PE groups. These tested antioxidant proteins could fall into three subclasses, including unchanged, up-regulated, and down-regulated, in response to implantation of both artificial joint and PE particles. The up-regulated proteins included transferrin, catalase, SOD3, GPx1 and TTR, whereas the down-regulated proteins contained SOD2 and GPx2 only (Fig 6). Levels of SOD1 were not significantly changed in knee joint tissues in response to the implantation of both artificial joint and PE particles (Fig 6). Results from Western blot analysis also revealed that inflammatory factors, NF-κB and IL-1β, and the key osteoclast-inducing factor RANKL were elevated at higher levels in the artificial joint/PE groups, when compare to control groups (Fig 6). Taken together, these results indicated that down-regulation of antioxidant enzyme expression is not a common event in the development of particle-induced oxidative damage.

## Discussion

Wear particles are known as the main causative agents in periprosthetic osteolysis [5]. Numerous studies have revealed that PE wear particles isolated from periprosthetic tissues are heterogeneous in sizes ranging from 0.1 μm to 1000 μm; however, the particles size distributions vary widely among reported studies [30, 31]. Although PE particles with the size range from 0.1 μm to 10 μm have the highest proportion of the wear volume of tissues samples, the large particles (> 10 μm) could account for 30% of the wear volume [32, 33]. Since wear particles in periprosthetic tissues are not uniform in sizes, host tissue responses may include phagocytosis of small particles (0.1–10 μm) by macrophages or engulfment of large particles (10–100 μm) by foreign body giant cells [34, 35]. Additionally, it could also be possible that large wear particles could be gradually broken down into small pieces mediated by macrophage-derived enzymes, a mechanism known as macrophage-mediated extracellular degradation of biomaterials [34]. Until now, it remains largely unclear whether large wear particles with the size range of 10–100 μm sufficiently initiate or/and promote inflammatory reactions and the subsequent osteolyitc process following a long-term persistence. In this study, we showed that implantation of large PE particles (20–60 μm) or artificial knee joint alone could not significantly affect levels of MDA and TAC in blood plasma during the 24-week experimental period, but the combination of an artificial joint and PE particles (1, 5 or 10 mg/ml) greatly increased MDA levels and decreased TAC levels, particularly during 20–24 weeks after operation. Importantly, higher PE exposure concentrations in the artificial joint implantation groups significantly contribute to more severe oxidative stress and inflammation, along with lower levels of physical activity (Figs 2–5). According to these findings, two important implications are proposed. First, the plasma MDA and TAC, two oxidative stress markers, could serve as important indicators for predicting the onset and/or progression of particle-induced chronic inflammation. Due to the potential influence of aging and other chronic inflammatory conditions on oxidative stress, the assessment of overall pain or local site pain may also need to be included in the prediction. Second, in addition to wear particles, other factors derived from the combination of artificial joint and wear particles may be critically required for particle-induced chronic inflammation.

**Fig 7:**
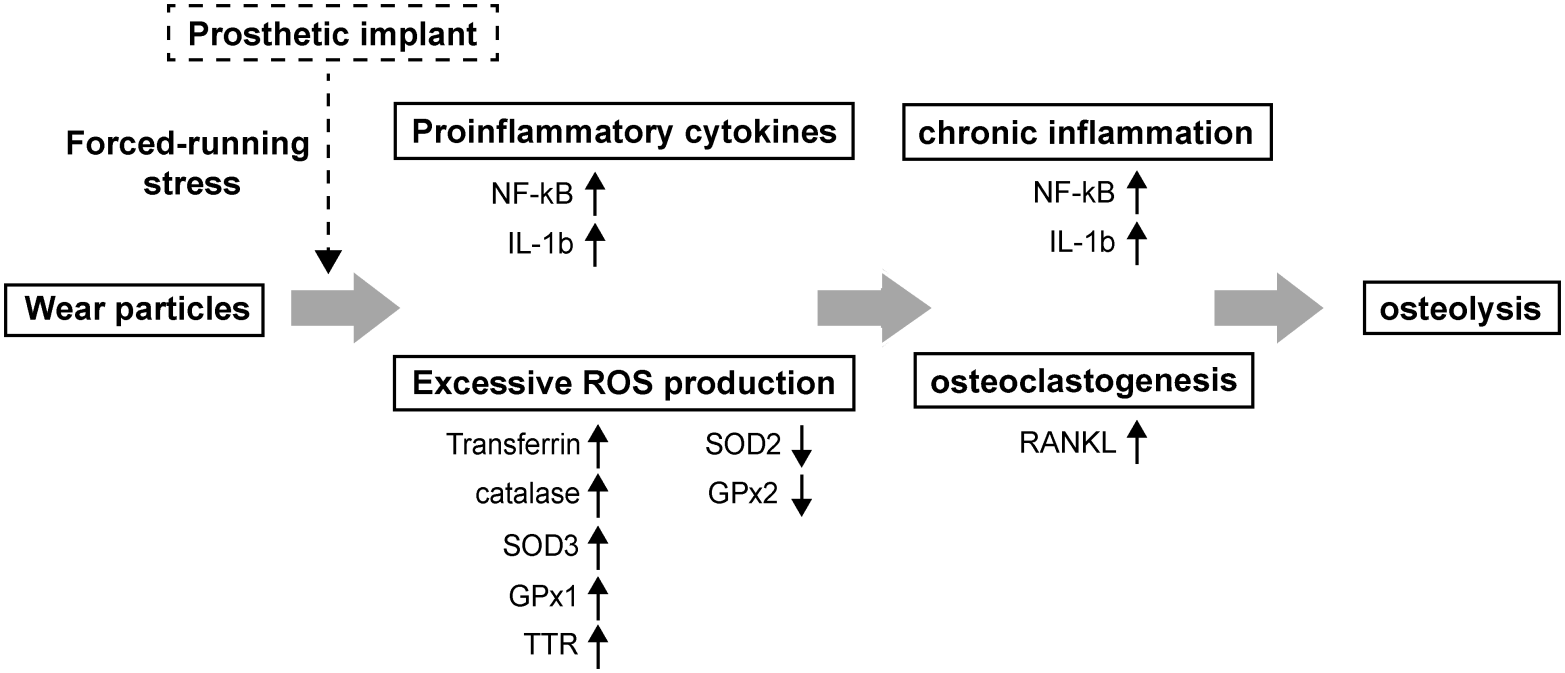
Proposed model for the biological and molecular events in the development of particle-induced periprosthetic osteolysis. Dysregulated expression of ROS-related proteins probably plays a critical role in the progression of particle-induced periprosthetic osteolysis.

Although we used large PE particles with the size of 20–60 μm in the study, our findings are generally consistent with previous studies using submicron PE particles. Sundfeldt et al. have previously shown that repeated injection of submicron PE particles every second week for up to 48 weeks in rabbits did not sufficiently induce osteolysis [36]. They conclude that wear particles are one, but not the only one etiology for aseptic loosening of joint implants [4, 36]. Additionally, in an *in vitro* experiments, Wang et al. showed that exposure of un-induced macrophages (THP-1) or osteoclasts to submicron-sized PE particles or zirconia particles was insufficient to stimulate the ROS release [10]. However, upon activation of these cells with TNF-α or PMA (phorbol-12-myristate-13-acetate), the ROS production under particulate stress was synergistically induced [10]. Therefore, prior activation of macrophages or osteoclasts by other stimuli may be essential for conferring cellular responses to wear particles. Although the detailed mechanisms are currently unknown, we do think that insertion of PE particles and artificial prosthesis in rat knee joints along with a long-term forced running stress may exert synergistic effects on oxidative damage, inflammatory reactions and the subsequent osteolysis around the prosthesis (Fig 7).

Oxidative stress reflects an imbalance between ROS production and ROS scavenging via multiple pro-oxidant and antioxidant systems. Previous studies have known that NADPH oxidase isoforms NOX1 and NOX2 were predominantly up-regulated in macrophages in response to particles [8, 9]. However, the expression of various antioxidant proteins during the development of particle-induced periprosthetic osteolysis is largely unknown. As expected, we here showed that implantation of artificial joint and PE particles in our rat model significantly promoted the expression of pro-inflammatory factors such as NF-κB and IL-1β as well as the osteoclast-inducing factor RANKL in periprosthetic tissues (Figs 5 and 6). Strikingly, although implantation of both artificial implants and PE particles down-regulated SOD2 and GPx2 in implanted tissues, the expressions of most other antioxidant proteins including transferrin, catalase, SOD3, GPx1 and TTR were actually up-regulated (Summary in Fig 7). Previously, Garrett et al. [20] have shown that excessive free radicals were closely associated with osteoclastogenesis induced by inflammatory factors such as IL-1 and parathyroid hormone (PTH). Interestingly, they found that the PTH- and IL-1-stimulated osteoclastic bone resorption was only inhibited by SOD, but not by catalase. In agreement with their reported data, we here demonstrated that the expression of SOD2, but not catalase, was substantially reduced in our animal model that received both PE particles and artificial knee joint. Notably, SOD2 is an essential mitochondrial antioxidant enzyme in protection against oxidative stress and cell apoptosis [37]. According to our findings, future SOD2-dependent therapeutic strategies may be important in limiting particle-induced oxidative stress and inflammation.

Despite the fact that an uncommon expression profile of antioxidant enzymes has been detected in periprosthetic tissues induced by large PE particles, our study has several limitations or unanswered questions worth noting. First, we here used large PE particles with the size range of 20–60 μm as starting materials in implantation studies. At present, we do not know whether the size distribution of implanted particles changes after a long-term persistence in rat joints, and whether foreign body giant cells play important roles in periprosthetic tissues associated with chronic inflammation and osteolysis. Second, due to several unexpected results from experiments, one weakness of our research designs is that several control groups, which receive only PE particles at different concentrations, are not included in the study. Third, it is currently unclear whether small particles (0.1–10 μm) produce the same outcomes as large particles (20–60 μm) shown in our established rat knee prosthesis model. Therefore, more research may be needed to further compare potential effects of large particles (10–100 μm), small particles (0.1–10 μm), and the combination of both on periprosthetic inflammation and osteolysis.

In summary, this study demonstrates that implantation of PE particles and artificial prosthesis in rat knee joints along with a forced running stress significantly trigger oxidative stress, chronic inflammation and osteolysis. Moreover, our findings show that down-regulation of antioxidant proteins is not a comment event in particle-induced oxidative stress. Therefore, it is possible that up-regulation or down-regulation in expression of specific antioxidant genes may be important for determining the progression of particle-induced periprosthetic inflammation and osteolysis.

## Supporting information

S1 Fig Photographs of the RAT 4CH FORCED EXERCISE system. The forced running wheel system is used to provide rats with a high-intensity exercise training and to assess levels of the physical activity. The wheel-running system is constructed with 32 cm in diameter, 8.5 cm in width, and 0.7 cm in the roller space.
(TIF)

S2 Fig Body weight of different treatment groups at different time points before and after operation. There are no significant differences in body weight among different treatment groups at pre-operation (Pre-OP), and at 4, 8, 16, and 24 weeks after operation.
(TIF)
