# Supplementary figures and images for: Dysregulated expression of antioxidant enzymes in polyethylene particle-induced periprosthetic inflammation and osteolysis
Source: PLoS One. 2018 Aug 20;13(8):e0202501. doi: 10.1371/journal.pone.0202501 (PMC6101395; doi:10.1371/journal.pone.0202501)

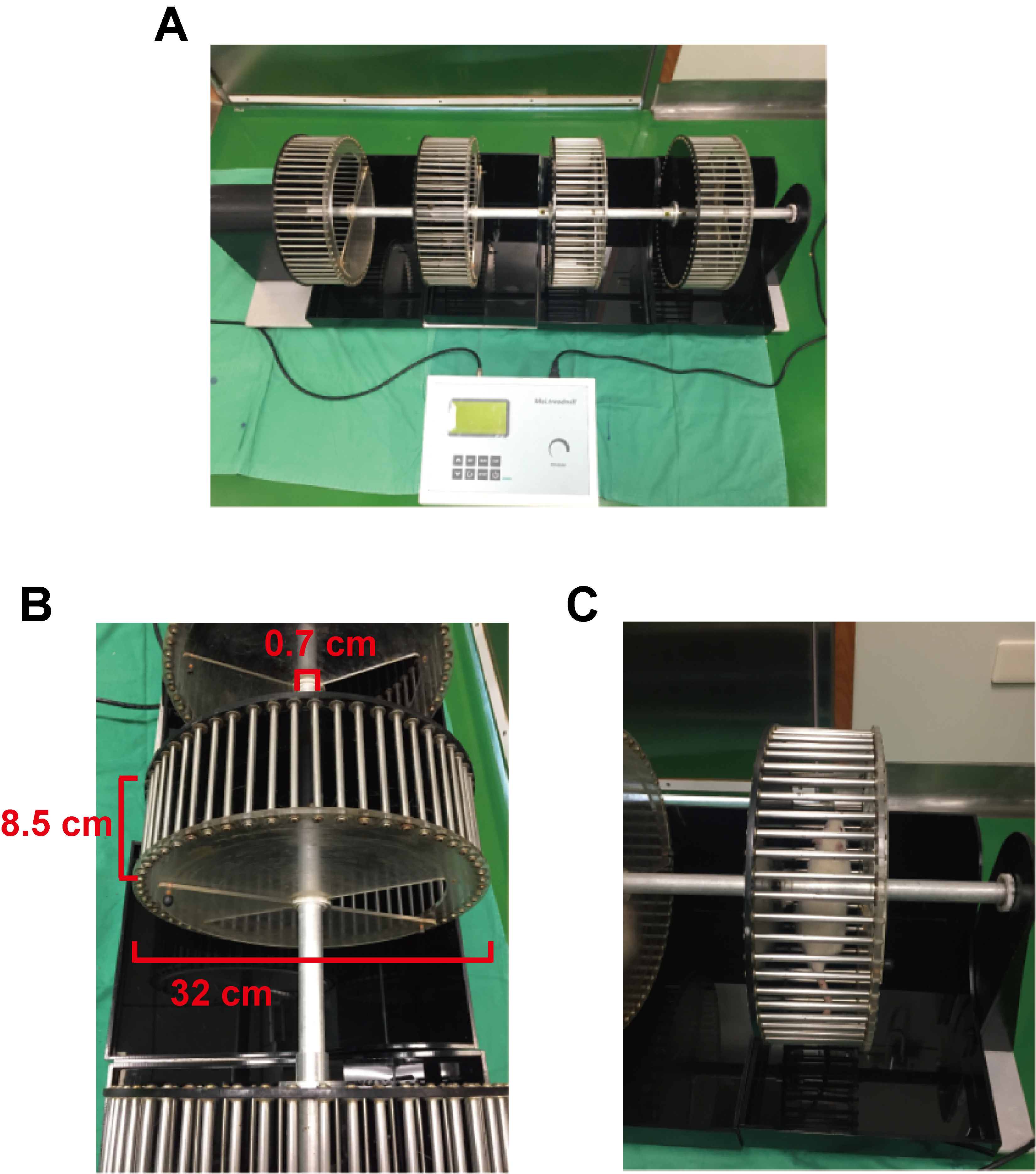

Supplement: S1 Fig — The forced running wheel system is used to provide rats with a high-intensity exercise training and to assess levels of the physical activity. The wheel-running system is constructed with 32 cm in diameter, 8.5 cm in width, and 0.7 cm in the roller space. (TIF) [file pone.0202501.s001.tif]

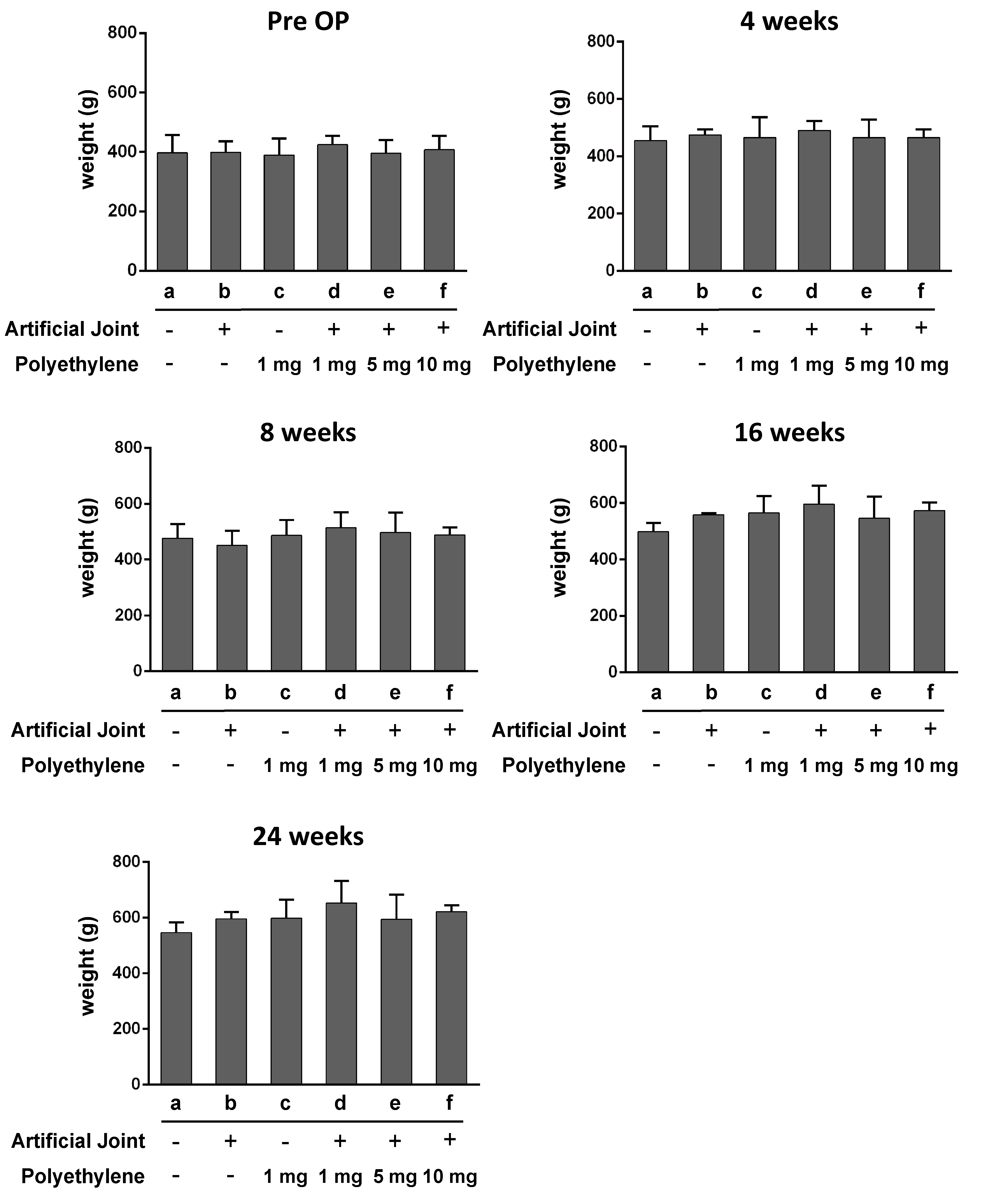

Supplement: S2 Fig — There are no significant differences in body weight among different treatment groups at pre-operation (Pre-OP), and at 4, 8, 16, and 24 weeks after operation. (TIF) [file pone.0202501.s002.tif]
